# Assessment of sub-chronic toxicity and anti-aging effects of a solid self-microemulsifying drug delivery system of *Kaempferia parviflora* extract in a D-galactose-induced rat model

**DOI:** 10.1080/13880209.2025.2606956

**Published:** 2026-01-05

**Authors:** Somsuda Somintara, Waraporn Sakaew, Sarunya Tuntiyasawasdikul, Peera Tabboon, Catheleeya Mekjaruskul, Jenjiralai Phanphak, Choowadee Pariwatthanakun, Bungorn Sripanidkulchai, Tawut Rudtanatip

**Affiliations:** a Electron Microscopy Unit, Department of Anatomy, Faculty of Medicine, Khon Kaen University, Khon Kaen, Thailand; b Faculty of Pharmaceutical Sciences, Khon Kaen University, Khon Kaen, Thailand; c Center for Research and Development of Herbal Health Products, Faculty of Pharmaceutical Sciences, Khon Kaen University, Khon Kaen, Thailand; d Integrative Pharmaceuticals and Innovation of Pharmaceutical Technology Research Unit, Faculty of Pharmacy, Mahasarakham University, Maha Sarakham, Thailand; e Division of Anatomy, Faculty of Medicine, Mahasarakham University, Maha Sarakham, Thailand

**Keywords:** *Kaempferia parviflora*, solid self-microemulsifying drug delivery system, sub-chronic toxicity, D-galactose, anti-aging

## Abstract

**Context:**

The pharmacological effects of *Kaempferia parviflora* have been extensively documented. A solid self-microemulsifying drug delivery system (SMEDDS) has been developed to address limitations such as poor water solubility and low bioavailability.

**Objective:**

This study evaluated the safety and anti-aging efficacy of the solid SMEDDS containing *K. parviflora* extract (KPS-SMEDDS) in rats.

**Materials and methods:**

A sub-chronic toxicity study was performed to assess the safety of KPS-SMEDDS. Healthy rats (*n* = 10/group) received oral doses of 125, 250, or 500 mg/kg body weight daily for 90 days. Clinical signs, body weight, hematological and biochemical parameters, and major organ histopathology were evaluated. Separately, the anti-aging effects of KPS-SMEDDS were investigated in a different cohort of rats (*n* = 9/group) with D-galactose-induced aging. Rats received intraperitoneal D-galactose (50 mg/kg/day) and oral KPS-SMEDDS at the same doses for 60 days. Oxidative stress markers, hormone levels, histopathology, and the expression of proteins related to aging, apoptosis, and inflammation were assessed.

**Results:**

No significant changes were observed in clinical signs, body weight, organ weights, hematological or biochemical parameters, or histopathology in KPS-SMEDDS-treated rats, indicating its safety. KPS-SMEDDS treatment significantly improved testicular weight, reduced malondialdehyde levels, normalized hormone levels, and restored testicular structure in rats with D-galactose-induced aging. Additionally, it upregulated SIRT-1 and Bcl-2, downregulated SA-β-gal, p53, and caspase-3, and modulated inflammatory cytokines (TNF-α, IL-6, and IL-10).

**Discussion and conclusion:**

KPS-SMEDDS was well-tolerated in rats and exerted protective effects against D-galactose-induced aging. These findings support its potential as a safe, natural anti-aging agent and highlight the value of formulation enhancement in traditional herbal medicine.

## Introduction

*Kaempferia parviflora* Wall. ex Baker (Zingiberaceae), a prominent herbal product of Thailand, is native to the northeastern region. It has been traditionally used in Thai medicine as a health-promoting tonic, particularly for enhancing physical performance, boosting vitality, and delaying the effects of aging (Wattanathorn et al. 2012; Yoshino et al. 2014). Traditionally regarded as an ‘elixir of life’, *K. parviflora* is believed to possess anti-fatigue, aphrodisiac, and longevity-promoting effects (Chaturapanich et al. 2008). The ethanol extract of its rhizome contains several methoxyflavones, including 3,5,7,3′,4′-pentamethoxyflavone (PMF), 5,7,4′-trimethoxyflavone (TMF), and 5,7-dimethoxyflavone (DMF) as major constituents (Sutthanut et al. 2007; Mekjaruskul et al. 2012; Tuntiyasawasdikul et al. 2024). Numerous studies have reported a wide range of pharmacological activities, including anti-inflammatory, anticancer, anti-allergic, anti-obesity, antidepressant, cognitive-enhancing, antidiabetic, and anti-aging effects (Elshamy et al. 2019; Klinngam et al. 2022). Recent scientific evidence increasingly supports these traditional claims, highlighting the potential of *K. parviflora* as a natural anti-aging agent and functional food ingredient for promoting healthy aging and overall well-being (Tuntiyasawasdikul and Sripanidkulchai 2022).

The oral route is the preferred method of drug delivery due to its convenience and high patient compliance. However, over 40% of orally administered drugs exhibit limited therapeutic efficacy because of poor aqueous solubility and absorption (Boyd et al. 2019). These challenges can be addressed through alternative formulation strategies, such as incorporating oils or fats to develop a solid self-microemulsifying drug delivery system (SMEDDS; Li et al. 2025).

SMEDDS is a liquid formulation consisting of surfactants, co-surfactants, and oils, into which a drug is incorporated. Upon contact with gastric fluid, it spontaneously forms an oil-in-water emulsion. Although thermodynamically unstable, SMEDDS enhances drug solubility and absorption. A SMEDDS formulation of *Kaempferia parviflora* extract (KPS-SMEDDS) was developed (Tuntiyasawasdikul et al. 2024); however, its sub-chronic toxicity and pharmacological properties, particularly its anti-aging activity, have not yet been investigated.

Aging is characterized by a gradual decline in biological functions. Various etiological factors contribute to aging, including both endogenous and environmental influences, with oxidative stress and inflammation being among the most significant (Tao et al. 2024). As the elderly population continues to rise globally, aging has become a major public health concern. Increasingly, individuals aim not only to extend life expectancy but also to prolong healthy, disease-free years. D-galactose has been widely used in research to induce accelerated aging in animal models (Aydin et al. 2018; Azman et al. 2021).

D-galactose is an aldohexose and a reducing sugar that occurs naturally in the body and many foods. Under normal conditions, it is efficiently metabolized and excreted. However, at elevated levels, it can be converted into aldose, hydroperoxide, and galactose oxidase, leading to the generation of reactive oxygen species (ROS). Mitochondria are a major source of intracellular ROS, which induce mitochondrial dysfunction and damage cellular macromolecules such as lipids, carbohydrates, proteins, and nucleic acids. This damage increases the production of inflammatory mediators, ultimately contributing to cellular aging and apoptosis (Azman and Zakaria 2019). Accumulation of ROS in cells and tissues promotes the development of age-associated diseases, including arthritis, cancer, type 2 diabetes, osteoporosis, hypertension, cardiovascular disease, and neurodegenerative disorders such as Parkinson’s and Alzheimer’s diseases (Liguori et al. 2018). Therefore, understanding the mechanisms of aging and identifying effective anti-aging therapies is of critical importance.

*K. parviflora* extract has been reported to inhibit cellular senescence and mitochondrial dysfunction in human dermal fibroblasts, alleviate aging symptoms in ultraviolet B (UVB)-irradiated mice, and extend the lifespan of *Caenorhabditis elegans* (Park et al. 2017; Tonsomboon et al. 2021). However, the safety and anti-aging efficacy of KPS-SMEDDS have not been clearly elucidated.

This study thus aimed to investigate the safety, specifically sub-chronic toxicity, and anti-aging effects of KPS-SMEDDS in a D-galactose-induced aging model in rats. Following oral administration of KPS-SMEDDS, sub-chronic toxicity and various aging-related parameters were evaluated, including biochemical and hormone levels, malondialdehyde (MDA) levels, cell morphology, and the expression of aging-associated proteins.

## Materials and methods

### Materials

The *K. parviflora* rhizomes used in this study were collected in Loei province, Thailand. Botanical identification was confirmed by a specialist, and a voucher specimen (HB 205/67) has been deposited at the Center for Research and Development of Herbal Health Products, Khon Kaen University. Reference standards of methoxyflavones – 98% DMF and 97% TMF – were purchased from Indofine Chemical Company Inc. (NJ, USA). The PMF standard was isolated from *K. parviflora* ethanolic extract *via* column chromatography following a previously described method (Tuntiyasawasdikul et al. 2024).

### Preparation of the plant extract

The ethanolic extract of *K. parviflora* was prepared by macerating the plant material in 95% ethanol for three days. After filtration, the plant residue was subjected to a second maceration, and all filtrates were pooled and concentrated using a rotary evaporator to obtain the oleoresin extract. The resulting extract had a yield of 4.75% and was stored at −20 °C until further use (Chankitisakul et al. 2025).

Due to the limited oral bioavailability of *K. parviflora* extract (Mekjaruskul et al. 2012), a solid self-microemulsifying drug delivery system containing *K. parviflora* extract (KPS-SMEDDS) was developed, adapted from a previous report (Tuntiyasawasdikul et al. 2024). Briefly, the ethanolic extract of *K. parviflora* was mixed with the oil phase (Neobee® M5), followed by gentle mixing with a surfactant (Cremophor EL) and a co-solvent (PEG 400). Using a geometric dilution method, the resulting mixture was blended in a 1:1 ratio with a silicon dioxide carrier (Sipernat® 22S). High-performance liquid chromatography analysis showed that the prepared KPS-SMEDDS contained the bioactive compounds PMF, DMF, and TMF at concentrations of 1.09–2.40%, 0.93–2.43%, and 1.06–2.23%, respectively.

### Experimental animals and housing conditions

Male and female Sprague Dawley rats (age, 6–8 weeks; weight, 280–300 g) were purchased from Nomura Siam International Co., Ltd. (Bangkok, Thailand). The animals were housed at an ambient temperature of 22 ± 2 °C with a 12-h light/dark cycle and acclimatized for 7 days prior to the experiment. Standard rat food (C.P. rat food 082S, W.T. Co., Ltd., Samutprakan, Thailand) and water were provided ad libitum.

All experiments were conducted in accordance with the National Institutes of Health Guide for the Care and Use of Laboratory Animals (NIH Publication No. 80-23, revised 1996). Standard procedures outlined in the Organization for Economic Cooperation and Development (OECD) Principles of Good Laboratory Practice, No. 408 (Repeated Dose 90-Day Oral Toxicity Study in Rodents; OECD, 1998), were followed. Animal use was approved by the Institutional Animal Care and Use Committee of Khon Kaen University (IACUC-KKU-141/64). All researchers complied with ARRIVE Guidelines 2.0. The experiments were conducted over a three-year period at the Northeast Laboratory Animal Center, the Faculty of Pharmaceutical Sciences, and the Faculty of Medicine, Khon Kaen University, between 1 January 2022 and 30 May 2025.

Animal inclusion and exclusion criteria: Healthy Sprague Dawley rats, aged 6–8 weeks and weighing 280–300 g, were included in the study. Animals that developed illness or injury unrelated to the experimental treatment, or whose biological samples were unsuitable for analysis (e.g., hemolyzed serum), were predefined for exclusion. No animals or datasets were excluded from the final analysis. Blinding was applied throughout the study to minimize observer and analytical bias.

### Sub-chronic toxicity evaluation of KPS-SMEDDS in rats

#### Clinical observation and examination

A total of 80 rats were used for the sub-chronic toxicity study and were randomly assigned into four groups (10 rats/sex/group): (1) control group (Control), (2) KPS-SMEDDS 125 mg/kg body weight group (KPS-SMEDDS 125), (3) KPS-SMEDDS 250 mg/kg body weight group (KPS-SMEDDS 250), and (4) KPS-SMEDDS 500 mg/kg body weight group (KPS-SMEDDS 500). Rats in the control group received distilled water, whereas those in the treatment groups received KPS-SMEDDS at the respective doses. All animals were weighed daily and received oral treatment for 90 days. Clinical signs, including mortality, were monitored and recorded once daily for each animal. Body weight was recorded at baseline, every three days thereafter, and at necropsy. Organ weights were measured at the end of the study following euthanasia.

#### Hematology and clinical biochemistry parameters

At the end of the experiment, rats were fasted overnight and euthanized *via* intraperitoneal injection of thiopental sodium (60 mg/kg body weight). Blood was collected from the inferior vena cava. Samples for hematological analysis were placed in tubes containing ethylenediaminetetraacetic acid (EDTA). Hematological parameters were analyzed using an automatic hematology analyzer (XS-800i; Sysmex Corporation, Japan). Measured parameters included red blood cell count (RBC), hemoglobin concentration (HGB), hematocrit (HCT), mean corpuscular volume (MCV), mean corpuscular hemoglobin (MCH), mean corpuscular hemoglobin concentration (MCHC), platelet count (PLT), and total white blood cell count (WBC), including differential counts of neutrophils, eosinophils, lymphocytes, monocytes, and basophils. For biochemical analyses, blood was collected in tubes without anticoagulant and centrifuged to obtain serum. Serum biochemistry parameters were measured using an automatic chemistry analyzer (Cobas Integra 400plus; Roche Diagnostics, Germany) and included alanine transaminase (ALT), aspartate transaminase (AST), gamma-glutamyl transferase (GGT), blood urea nitrogen (BUN), creatinine, cholesterol, triglycerides, high-density lipoprotein (HDL)-cholesterol, low-density lipoprotein (LDL)-cholesterol, and hemoglobin A1c (HbA1c).

#### Necropsy and histopathological examination

At termination, various organs – including the brain, heart, lungs, liver, kidneys, spleen, stomach, intestine, testis, and ovaries – were harvested, rinsed with cold normal saline, blotted dry with filter paper, and weighed to calculate the organ index. The organ index was calculated using the formula: [organ weight (g)/body weight (g)] × 100. Collected organs were processed for histological examination using hematoxylin and eosin staining. Tissues were fixed in 10% phosphate-buffered formalin (pH 7), embedded in paraffin, sectioned at 5 µm thickness, and stained. Tissue morphology was examined under a light microscope (Olympus, Tokyo, Japan).

### Effect of KPS-SMEDDS against D-galactose induced-aging in rats

#### Animal treatment and study design

A total of 63 male rats were used for the D-galactose–induced aging study and were randomly divided into seven groups (9 rats/group): (1) control group (Control), (2) D-galactose group (D-gal), (3) D-galactose + vehicle group (D-gal + Veh), (4) D-galactose + KPS-SMEDDS 125 mg/kg body weight group (D-gal + KPS-SMEDDS 125), (5) D-galactose + KPS-SMEDDS 250 mg/kg body weight group (D-gal + KPS-SMEDDS 250), (6) D-galactose + KPS-SMEDDS 500 mg/kg body weight group (D-gal + KPS-SMEDDS 500), and (7) D-galactose + resveratrol 50 mg/kg body weight group (D-gal + Res). Rats in groups 2–7 received intraperitoneal injections of D-galactose (50 mg/kg/day; Sigma Aldrich, St. Louis, MO, USA) dissolved in 0.9% normal saline for 60 days, while the control group received an equal volume of normal saline on the same schedule. Rats in the KPS-SMEDDS and resveratrol groups were administered KPS-SMEDDS (dissolved in distilled water) at doses of 125, 250, and 500 mg/kg, or resveratrol at 50 mg/kg, respectively, *via* intragastric gavage for 60 days. Resveratrol was included as a positive anti-aging control due to its antioxidant, anti-inflammatory, and anti-degenerative properties (Perrone et al. 2017). Late-life resveratrol treatment has been shown to extend lifespan and mitigate age-related symptoms in several model organisms (Gao et al. 2023). The resveratrol solution contained the following components (% w/w): 5% resveratrol, 28% propylene glycol, 35% PEG 400, 2% ethanol, and 30% deionized water.

All animals were monitored biweekly for body weight and general health. At the end of the experiment, rats were euthanized under deep anesthesia with thiopental sodium (60 mg/kg body weight, intraperitoneally). Brain, liver, kidney, and testis functions gradually decline with age, primarily due to ROS–induced damage, and aging is associated with marked cellular changes in these organs (Tenchov et al. 2024). Therefore, blood, brains, livers, kidneys, and testes were collected for further analysis. Each organ was immediately removed, weighed, and divided into three parts: the first was minced and ground in liquid nitrogen for Western blot analysis; the second was fixed in 10% buffered formalin at room temperature for histopathological examination; and the third was fixed in 2% glutaraldehyde for ultrastructural studies.

#### Biochemical and hormone determination

At the end of the treatment period, rats were fasted overnight and euthanized *via* intraperitoneal injection of thiopental sodium (60 mg/kg body weight). Blood was collected from the inferior vena cava. For biochemical and hormone analyses, blood samples were collected in tubes without anticoagulant and centrifuged to obtain serum. Serum parameters were measured using an automatic chemistry analyzer (Cobas Integra 400plus; Roche Diagnostics, Germany) and included ALT, AST, BUN, creatinine, cholesterol, triglycerides, HDL-cholesterol, LDL-cholesterol, HbA1c, follicle-stimulating hormone (FSH), luteinizing hormone (LH), and testosterone.

#### Measurement of malondialdehyde levels

Lipid peroxidation, expressed as malondialdehyde (MDA) content, was measured using a commercial MDA assay kit (Thiobarbituric Acid Reactive Substances; Sigma-Aldrich, St. Louis, MO, USA; Cat. No. MAK568) following the manufacturer’s instructions. Briefly, tissue samples (10 mg) were homogenized on ice in 300 µL of MDA lysis buffer and centrifuged at 13,000 × g for 10 min to remove insoluble material. Then, 200 µL of the supernatant was mixed with 600 µL of thiobarbituric acid solution, incubated at 95 °C for 60 min, and cooled in an ice bath for 10 min. Subsequently, 200 µL of the reaction mixture was transferred to 96-well black plates with clear bottoms, and absorbance was measured at 532 nm using a Varioskan LUX multimode microplate reader (Thermo Scientific, USA). MDA levels were expressed as nmol/ml of protein, and all analyses were performed in triplicate.

#### Histological analysis

Brain, liver, kidney, and testis tissue samples were fixed in 10% (w/v) buffered formalin for 24 h. Samples were processed through a graded alcohol series, cleared in xylene, and embedded in paraffin using a standard protocol. Sections of 5 µm thickness were stained with hematoxylin and eosin and examined under a light microscope (Olympus, Tokyo, Japan).

#### Electron microscopic examination

Because histological analysis showed that KPS-SMEDDS significantly reversed degenerative changes in the testes and increased testosterone levels, the testes were selected for further investigation. Fresh testis tissue samples from each group were fixed in 2% glutaraldehyde for 24 h and post-fixed in 1% osmium tetroxide in 0.1 M phosphate buffer for 1 h. Tissues were washed three times with PBS and dehydrated through a graded ethanol series (50%, 70%, 80%, 90%, and 95% for 5 min each, followed by 100% for 10 min). Samples were embedded in Epon 812 overnight. Ultrathin sections were prepared using an Ultracut N ultramicrotome (Reichert-Nissei, Tokyo, Japan), stained with 5% uranyl acetate and 2% lead citrate, and examined under a JEM-1010 transmission electron microscope (JEOL, Tokyo, Japan).

#### Western blot analysis

Total protein from testis tissue was extracted using RIPA lysis buffer supplemented with 100× protease inhibitor solution. Protein concentration was determined using a NanoDrop 2000 spectrophotometer (Thermo Scientific, USA). Equal amounts of total protein (30 µg) from each group were separated on a 12.5% SDS-polyacrylamide gel and transferred to a nitrocellulose membrane (Merck, Germany). Membranes were blocked with 2% BSA in TBS-T (100 mM Tris-base, 150 mM NaCl, 0.1% Tween 20) for 2 h, then incubated overnight at 4 °C with primary antibodies specific (1:1,000 dilution) for mouse-anti-Sirt-1 (cat. no. 8469S, Cell Signaling Technology Inc., USA), rabbit-anti-SA-β-gal (cat. no. 27198S, Cell Signaling Technology Inc., USA), rabbit-anti-TNF-α (cat. no. 3707S, Cell Signaling Technology Inc., USA), mouse-anti-IL-6 (cat. no. 12019S, Cell Signaling Technology Inc., USA), rabbit-anti-IL-10 (cat. no. 12163S, Cell Signaling Technology Inc., USA), mouse-anti-Bcl-2 (cat. no. 15071S, Cell Signaling Technology Inc., USA), rabbit-anti-caspase-3 (cat. no. MA5-35335, Invitrogen, Thermo Fisher Scientific, USA), mouse-anti-p53 (cat. no. 2524S, Cell Signaling Technology Inc., USA) and rabbit anti-β-actin (cat. no. AF7018, Affinity Biosciences, USA). After two washes with TBS-T, membranes were incubated with HRP-conjugated secondary antibodies (1:2,000 dilution; cat. no. 31460 for anti-rabbit antibody and cat. no. 626520 for anti-mouse antibody; Invitrogen, Thermo Fisher Scientific, USA) for 2 h at room temperature. Immunoreactive bands were visualized using Clarity™ Western ECL substrate (Bio-Rad Laboratories, USA). Protein expression levels were normalized to β-actin as an internal control and reported as fold changes relative to the control. The membranes were photographed and analyzed using ImageJ software (http://rsbweb.nih.gov/ij/download.html).

### Statistical analysis

All graphs, calculations, and statistical analyses were performed using GraphPad Prism version 9.0 for Mac (GraphPad Software, San Diego, CA, USA). Data were analyzed using one-way ANOVA followed by Tukey’s post hoc test. Results are presented as mean ± SEM, and differences were considered statistically significant at *p* < 0.05.

## Results

### KPS-SMEDDS exhibited no toxicity in rats under sub-chronic exposure

#### Body and organ weights of rats after KPS-SMEDDS administration

**Figure 1.**
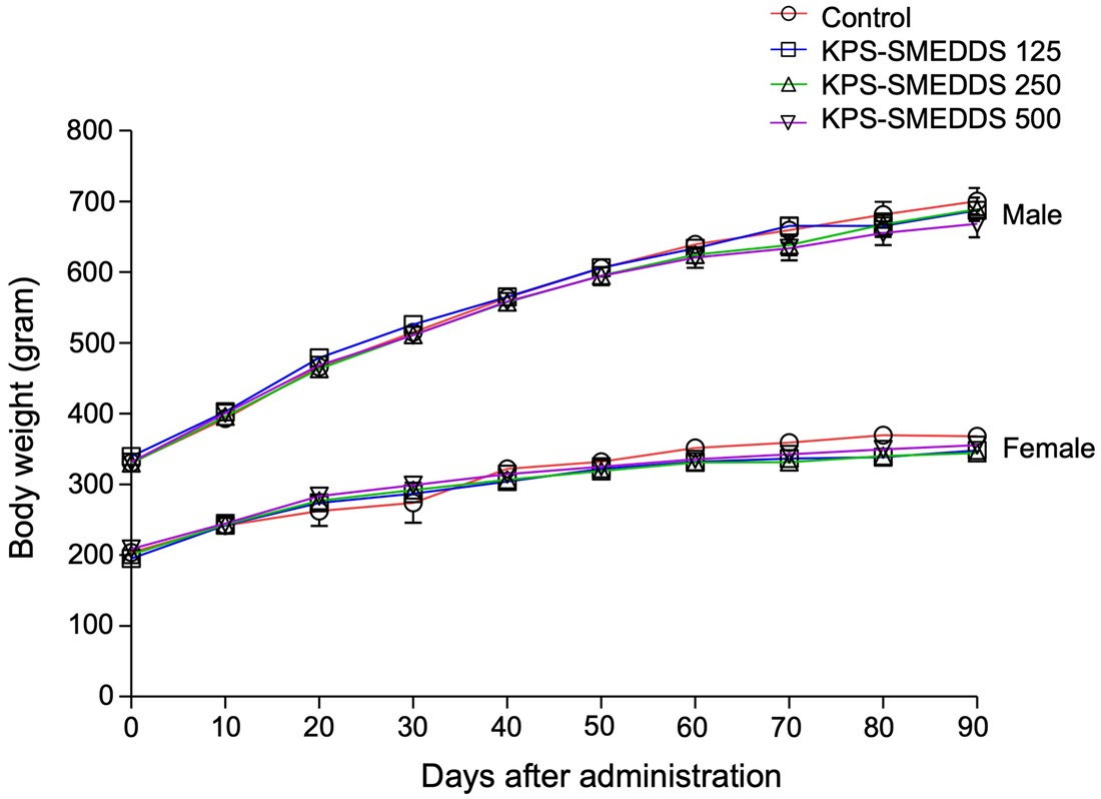
Growth curves of male and female rats in the sub-chronic toxicity study of KPS-SMEDDS. Growth curves of male and female rats orally administered KPS-SMEDDS at doses of 125, 250, and 500 mg/kg body weight once daily for 90 days. Body weight was recorded biweekly throughout the study period. The control group received distilled water only. Data are expressed as mean ± SEM (*n* = 10 per sex per group). Statistical analyses were performed using one-way ANOVA followed by Tukey’s post hoc test to compare each treatment group with the control. Significant differences were considered at *p* < 0.05. This figure demonstrates that repeated oral administration of KPS-SMEDDS for 90 days did not cause abnormal changes in body weight in either male or female rats, suggesting no adverse effects on growth under sub-chronic exposure conditions.

Based on acute toxicity testing, the approximate lethal dose of KPS-SMEDDS extract was greater than 5000 mg/kg body weight in both male and female Sprague Dawley rats (Tuntiyasawasdikul et al. 2024). Therefore, doses of 125, 250, and 500 mg/kg body weight were selected for the sub-chronic toxicity study. No deaths or treatment-related clinical signs were observed in any KPS-SMEDDS-treated group. Additionally, no gross lesions were detected in any organs at necropsy. There were no significant differences in daily body weight between the treatment and control groups (Figure 1). Relative organ weights, as presented in Table 1, showed no significant changes associated with KPS-SMEDDS extract in either sex at any dose level compared with their respective control groups.

**Table 1.** Relative organ weights (%) in rats following oral administration of KPS-SMEDDS. Rats were administered KPS-SMEDDS at doses of 125, 250, or 500 mg/kg body weight daily for 60 days.

Parameters	Dose of KPS-SMEDDS (mg/kg of body weight)							
	Males				Females			
	Control	125	250	500	Control	125	250	500
Liver	3.12 ± 0.16	3.14 ± 0.05	3.15 ± 0.04	3.12 ± 0.06	3.83 ± 0.12	3.96 ± 0.09	3.79 ± 0.08	3.74 ± 0.12
Spleen	0.14 ± 0.01	0.15 ± 0.01	0.15 ± 0.01	0.15 ± 0.01	0.19 ± 0.01	0.20 ± 0.02	0.19 ± 0.02	0.19 ± 0.01
Kidney (both sides)	0.61 ± 0.03	0.65 ± 0.01	0.61 ± 0.01	0.66 ± 0.02	0.71 ± 0.03	0.70 ± 0.04	0.73 ± 0.02	0.74 ± 0.03
Stomach	0.51 ± 0.02	0.54 ± 0.02	0.55 ± 0.03	0.54 ± 0.02	0.82 ± 0.03	0.85 ± 0.03	0.81 ± 0.04	0.81 ± 0.05
Intestine	4.74 ± 0.14	4.60 ± 0.10	4.61 ± 0.11	4.78 ± 0.11	6.65 ± 0.28	7.01 ± 0.08	6.78 ± 0.33	6.68 ± 0.21
Lung (both sides)	0.37 ± 0.02	0.39 ± 0.01	0.36 ± 0.01	0.38 ± 0.01	0.51 ± 0.02	0.57 ± 0.04	0.52 ± 0.05	0.53 ± 0.01
Brain	0.25 ± 0.01	0.26 ± 0.01	0.25 ± 0.01	0.27 ± 0.01	0.42 ± 0.02	0.43 ± 0.01	0.45 ± 0.00	0.44 ± 0.01
Heart	0.29 ± 0.02	0.29 ± 0.01	0.28 ± 0.01	0.30 ± 0.01	0.38 ± 0.02	0.39 ± 0.01	0.39 ± 0.01	0.40 ± 0.02
Testis (both sides) in male or ovary (both sides) in female	0.54 ± 0.03	0.55 ± 0.01	0.55 ± 0.02	0.58 ± 0.02	0.32 ± 0.03	0.30 ± 0.02	0.28 ± 0.02	0.32 ± 0.02

Relative organ weight was calculated as the ratio of organ weight to body weight and expressed as mean ± SEM for both male and female rats (*n* = 10 per sex per group). Organs assessed included liver, spleen, stomach, intestine, lung, brain, heart, and reproductive organs. Statistical analysis was performed using one-way ANOVA followed by Tukey’s post hoc test. *p < 0.05 indicates a significant difference compared with the control group.

#### Hematology and biochemistry parameters of rats after KPS-SMEDDS administration

No statistically significant changes in hematological parameters were observed following KPS-SMEDDS administration (Table 2). Biochemical analysis revealed that cholesterol levels in male rats treated with the high dose of KPS-SMEDDS (500 mg/kg body weight) were significantly lower than those in the control group. Triglyceride levels were also significantly reduced in both male and female rats receiving 500 mg/kg body weight. LDL-cholesterol levels in male rats showed statistically significant reductions in both the medium-dose (250 mg/kg body weight) and high-dose (500 mg/kg body weight) groups, indicating a dose-dependent effect. Although HDL-cholesterol levels did not differ significantly at any dose, a dose-dependent trend was observed (Table 3).

**Table 2.** Hematological parameters in male and female rats following oral administration of KPS-SMEDDS. Rats received KPS-SMEDDS at doses of 125, 250, or 500 mg/kg body weight daily for 60 days.

Parameters	Dose of KPS-SMEDDS (mg/kg of body weight)							
	Males				Females			
	Control	125	250	500	Control	125	250	500
RBC (x10^6^ cells/µL)	9.48 ± 0.08	9.16 ± 0.20	9.23 ± 0.12	9.37 ± 0.13	8.27 ± 0.08	8.24 ± 0.14	8.40 ± 0.11	8.42 ± 0.09
Hemoglobin (g/dL)	15.94 ± 0.12	15.33 ± 0.24	15.46 ± 0.18	15.79 ± 0.16	14.68 ± 0.18	14.31 ± 0.22	14.69 ± 0.12	14.70 ± 0.11
Hematocrit (%)	48.57 ± 0.38	46.93 ± 0.52	47.58 ± 0.43	48.21 ± 0.34	47.48 ± 0.59	46.03 ± 0.48	47.83 ± 0.41	47.60 ± 0.48
MCV (fL)	51.26 ± 0.56	51.36 ± 0.68	51.59 ± 0.48	51.51 ± 0.61	57.43 ± 0.70	55.89 ± 0.66	56.98 ± 0.89	56.56 ± 0.60
MCH (pg)	16.83 ± 0.13	16.76 ± 0.15	16.75 ± 0.19	16.86 ± 0.13	17.74 ± 0.22	17.38 ± 0.16	17.50 ± 0.18	17.48 ± 0.17
MCHC (g/dL)	32.82 ± 0.15	32.67 ± 0.20	32.50 ± 0.19	32.74 ± 0.20	30.92 ± 0.10	31.09 ± 0.23	30.71 ± 0.22	30.89 ± 0.11
Platelets (x10^3^cells/µL)	1026.90 ± 29.33	1122.67 ± 60.99	1049.13 ± 36.65	1030.00 ± 38.47	969.30 ± 33.81	1004.38 ± 90.93	1055.75 ± 34.37	1010.10 ± 19.59
WBC (x10^3^ cells/µL)	9.41 ± 0.49	10.05 ± 1.27	8.67 ± 0.40	7.29 ± 0.44	5.99 ± 0.24	5.32 ± 0.50	5.13 ± 0.36	4.13 ± 0.20
Neutrophils (%)	8.62 ± 0.39	10.01 ± 0.51	10.62 ± 0.39	10.08 ± 0.34	9.59 ± 1.33	9.80 ± 0.73	10.58 ± 0.59	10.65 ± 0.35
Lymphocytes (%)	86.43 ± 0.95	84.70 ± 0.71	82.48 ± 0.95	82.10 ± 1.11	84.15 ± 1.36	84.18 ± 1.25	83.60 ± 0.50	82.31 ± 0.65
Monocytes (%)	3.94 ± 0.41	4.18 ± 0.39	4.79 ± 0.34	4.20 ± 0.24	3.06 ± 0.20	3.08 ± 0.20	2.73 ± 0.35	2.69 ± 0.26
Eosinophils (%)	1.79 ± 0.14	2.13 ± 0.31	2.09 ± 0.14	2.34 ± 0.20	1.51 ± 0.12	1.95 ± 0.32	2.14 ± 0.32	2.21 ± 0.14
Basophils (%)	0.01 ± 0.01	0.00 ± 0.00	0.01 ± 0.01	0.00 ± 0.00	0.14 ± 0.08	0.10 ± 0.07	0.10 ± 0.06	0.15 ± 0.11

The values are expressed as mean ± SEM (*n* = 10 per group) for both sexes. Parameters measured included white blood cell count (WBC), red blood cell count (RBC), hemoglobin (HGB), hematocrit (HCT), mean corpuscular volume (MCV), mean corpuscular hemoglobin (MCH), mean corpuscular hemoglobin concentration (MCHC), platelet count (PLT), and differential leukocyte counts (neutrophils, lymphocytes, monocytes, eosinophils, basophils). Statistical significance was determined using one-way ANOVA followed by Tukey’s post hoc test. *p < 0.05 indicates a significant difference compared with the control group.

**Table 3.** Biochemical parameters in male and female rats following oral administration of KPS-SMEDDS. Rats received KPS-SMEDDS at doses of 125, 250, or 500 mg/kg body weight daily for 60 days.

	Dose of KPS-SMEDDS (mg/kg of body weight)							
Parameters	Males				Females			
	Control	125	250	500	Control	125	250	500
ALT (U/L)	27.00 ± 0.95	24.44 ± 0.67	28.00 ± 1.97	25.30 ± 1.56	31.40 ± 1.32	30.00 ± 1.56	31.22 ± 1.90	29.50 ± 1.10
AST (U/L)	190.60 ± 5.67	196.78 ± 10.27	198.25 ± 6.76	178.70 ± 8.96	196.90 ± 9.21	174.00 ± 8.02	192.67 ± 11.01	195.50 ± 8.91
Gamma-GT (U/L)	3.00 ± 0.00	3.00 ± 0.00	3.00 ± 0.00	3.00 ± 0.00	3.00 ± 0.00	3.00 ± 0.00	3.00 ± 0.00	3.00 ± 0.00
BUN (mg/dL)	19.50 ± 0.43	19.22 ± 0.64	21.25 ± 0.53	19.30 ± 0.37	25.20 ± 1.04	23.38 ± 1.52	24.67 ± 1.11	23.70 ± 0.91
Creatinine (mg/dL)	0.32 ± 0.01	0.34 ± 0.02	0.36 ± 0.02	0.38 ± 0.01	0.38 ± 0.01	0.33 ± 0.02	0.36 ± 0.02	0.35 ± 0.02
HbA1C (%)	4.22 ± 0.03	4.19 ± 0.05	4.16 ± 0.02	4.12 ± 0.03	4.06 ± 0.04	4.03 ± 0.03	3.91 ± 0.06	4.03 ± 0.03
Cholesterol (mg/dL)	73.50 ± 2.20	73.33 ± 2.14	68.88 ± 1.43	61.80 ± 3.04*	87.80 ± 4.35	76.75 ± 3.05	77.22 ± 3.73	82.20 ± 4.72
Triglyceride (mg/dL)	393.40 ± 43.15	309.33 ± 46.60	255.50 ± 21.52	210.90 ± 21.72*	397.88 ± 36.88	290.29 ± 39.94	268.63 ± 45.26	188.63 ± 16.09*
HDL-Cholesterol (mg/dL)	48.50 ± 2.03	47.44 ± 2.24	46.25 ± 1.25	42.43 ± 1.21	67.20 ± 3.46	59.50 ± 3.97	60.67 ± 2.70	66.30 ± 4.27
LDL-Cholesterol (mg/dL)	7.30 ± 0.56	7.22 ± 0.36	6.88 ± 0.35*	6.50 ± 0.45*	7.40 ± 0.31	7.25 ± 0.39	7.00 ± 0.25	7.00 ± 0.39

The values are presented as mean ± SEM (*n* = 10 per group) for both sexes. Parameters measured included liver function markers (alanine aminotransferase [ALT], aspartate aminotransferase [AST]), kidney function markers (creatinine, blood urea nitrogen [BUN]), and lipid profile (cholesterol, triglycerides, high-density lipoprotein [HDL], low-density lipoprotein [LDL]). Statistical significance was determined using one-way ANOVA followed by Tukey’s post hoc test. *p < 0.05 indicates a significant difference compared with the control group, while #*p* < 0.05 indicates a significant difference compared with the D-galactose-induced group.

#### Histopathological features of rats after KPS-SMEDDS administration

**Figure 2.**
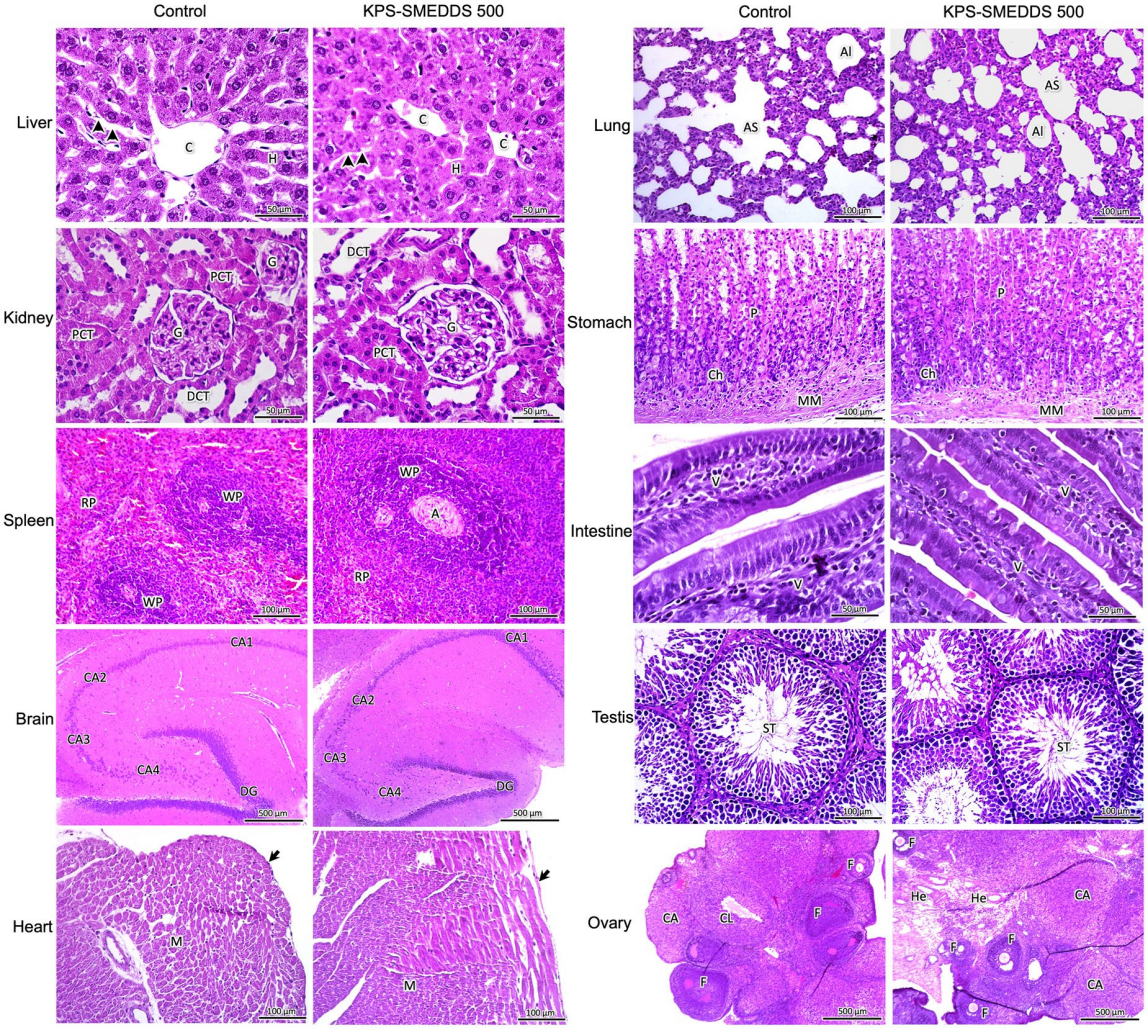
Histopathological assessment of major organs in rats treated orally with a high dose of KPS-SMEDDS (500 mg/kg body weight) for 90 days. Photomicrographs of liver, kidney, spleen, brain, heart, lung, stomach, intestine, and testis represent tissues from male rats, while photomicrographs of the ovary represent tissues from female rats. All tissue sections were stained with H&E. A: arteriole; Al: alveoli; AS: alveolar sac; C: central vein; CA: corpus albicans; CA1–CA4: cornu Ammonis 1–4; Ch: chief cells; CL: corpus luteum; DCT: distal convoluted tubule; DG: dentate gyrus; F: follicle; G: glomerulus; H: hepatic cord; He: helicine arteries; M: myocardium; MM: muscularis mucosae; P: parietal cells; PCT: proximal convoluted tubule; RP: red pulp; ST: seminiferous tubule; WP: white pulp; V: intestinal villi; arrowheads: hepatic sinusoid; arrows: epicardium. Scale bars = 50 μm, 100 μm, 500 μm.

Necropsy revealed no remarkable gross lesions in any organs of the KPS-SMEDDS–treated or control groups. Histopathological examinations of the liver, kidney, spleen, brain, stomach, intestine, heart, lung, testis, and ovary in rats are shown in Figures 2 and S1. Microscopic observation of these organs in KPS-SMEDDS-treated rats revealed normal structural architecture, comparable to that of the control group. These findings indicate that KPS-SMEDDS does not exhibit sub-chronic toxicity in this animal model.

### KPS-SMEDDS mitigated aging effects induced by D-galactose in rats

#### Body and organ weights of D-galactose-induced aging rats following KPS-SMEDDS administration

**Figure 3.**
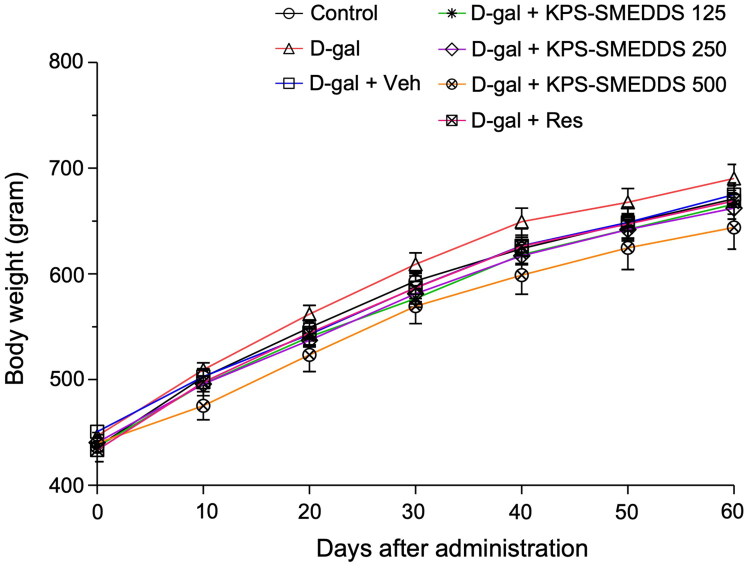
Growth curves of D-galactose–induced aging rats treated with KPS-SMEDDS. Growth curves of rats subjected to D-galactose–induced aging and orally administered KPS-SMEDDS at doses of 125, 250, and 500 mg/kg body weight for 60 days. Body weight was recorded biweekly throughout the experimental period. The control group received vehicle only, while the D-gal group received D-galactose without KPS-SMEDDS. Data are presented as mean ± SEM (*n* = 9 per group). Statistical comparisons were performed using one-way ANOVA followed by Tukey’s post hoc test. A significant difference was considered at *p* < 0.05. This figure illustrates that KPS-SMEDDS administration did not produce adverse effects on body weight and tended to preserve normal growth patterns in D-galactose-induced aging rats.

**Figure 4.**
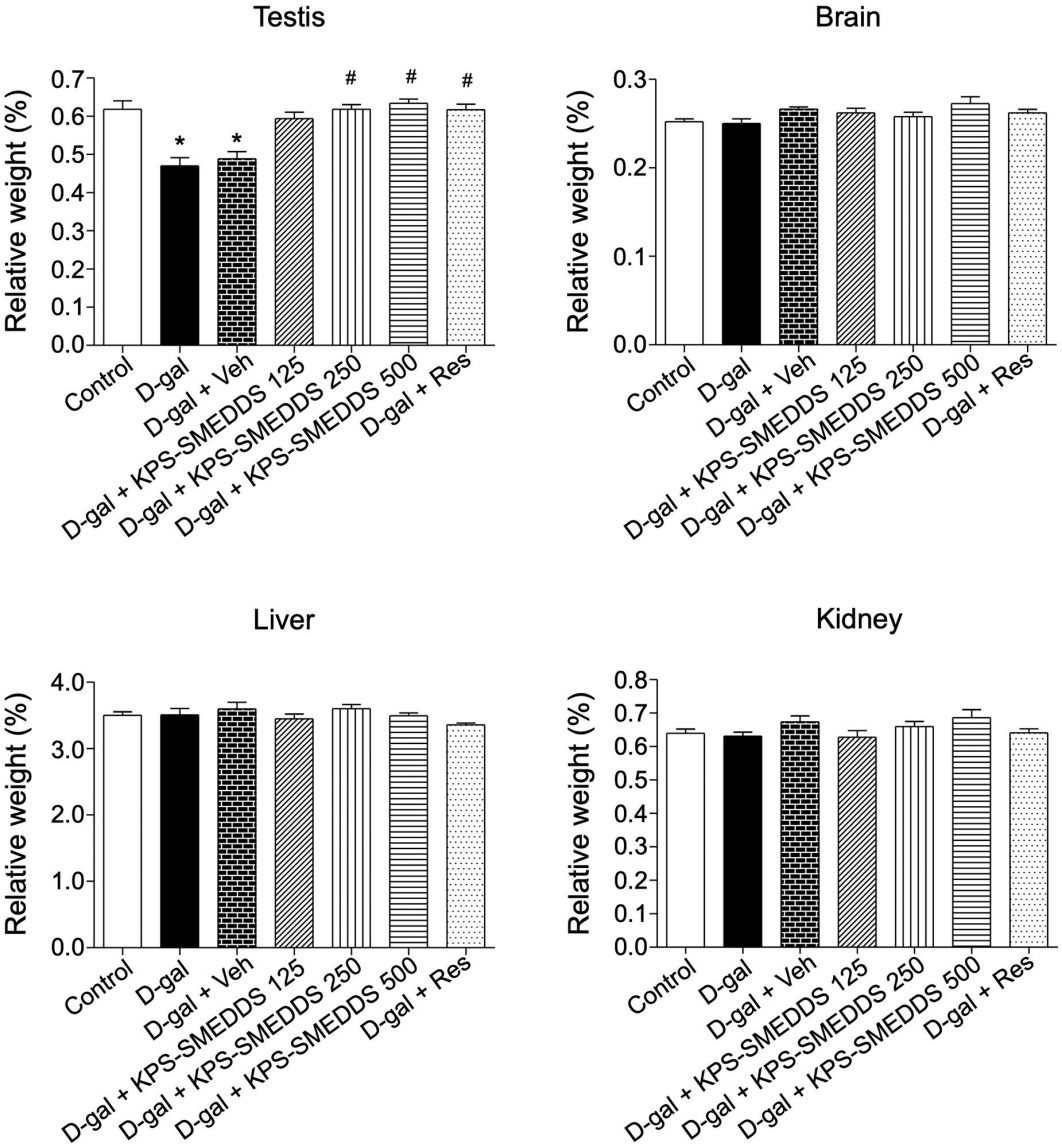
Relative organ weights in D-galactose-induced aging rats treated with KPS-SMEDDS. Rats were administered D-galactose to induce aging and subsequently received KPS-SMEDDS orally at doses of 125, 250, or 500 mg/kg body weight for 60 days. Relative organ weights were calculated as the ratio of organ weight to body weight and are presented as mean ± SEM (*n* = 9 per group). Statistical significance was determined using one-way ANOVA followed by Tukey’s post hoc test. *p < 0.05 indicates a significant difference compared with the normal control group, while #*p* < 0.05 indicates a significant difference compared with the D-galactose-induced group. Organs analyzed included the testis, brain, liver, and kidney.

No statistically significant differences in body weight changes were observed between the KPS-SMEDDS–treated groups and the control group. No deaths or treatment-related clinical signs occurred at any KPS-SMEDDS dose level, and no gross lesions were detected in any organs at necropsy. Additionally, there were no significant differences in daily body weight between treatment and control groups (Figure 3). Relative organ weight analysis showed that the D-galactose-induced aging groups (D-gal and D-gal + Veh) exhibited significantly decreased testicular weights compared to the control group. In contrast, rats treated with 250 and 500 mg/kg body weight of KPS-SMEDDS or 50 mg/kg body weight of resveratrol showed significantly increased testicular weights, comparable to normal controls. No statistically significant differences were observed among groups in the relative weights of the brain, kidneys, or liver (Figure 4).

#### Effects of KPS-SMEDDS on clinical biochemical and hormonal levels in D-galactose-induced aging rats

**Figure 5.**
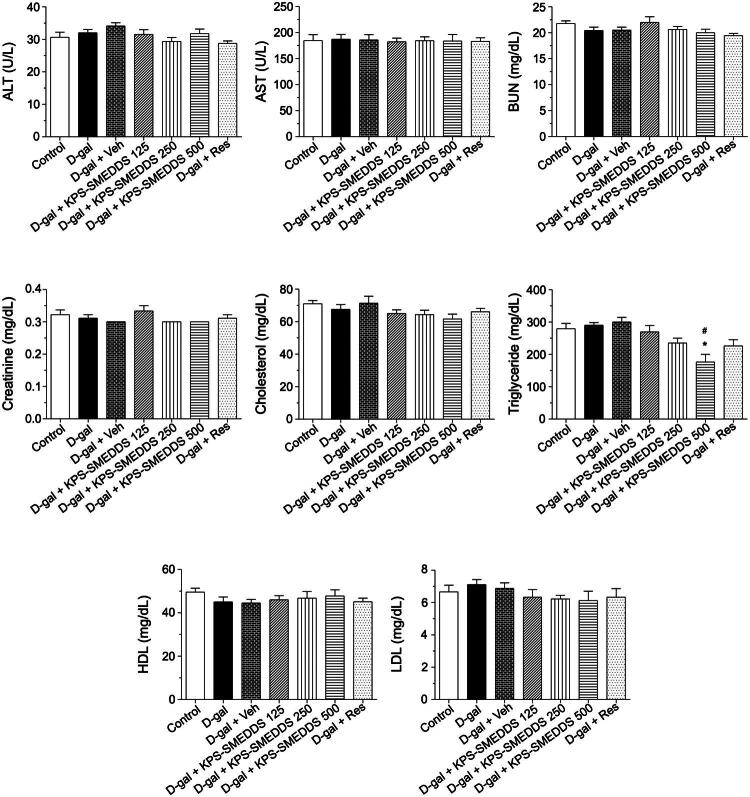
Effect of KPS-SMEDDS on serum biochemical parameters in D-galactose-induced aging rats. Rats were administered D-galactose to induce aging and subsequently treated with KPS-SMEDDS orally at doses of 125, 250, or 500 mg/kg body weight for 60 days. Serum biochemical parameters, including liver enzymes (ALT, AST), kidney function markers (creatinine, BUN), and lipid profile (cholesterol, triglycerides, HDL, LDL), were measured and are presented as mean ± SEM (*n* = 9 per group). Statistical significance was determined by one-way ANOVA followed by Tukey’s post hoc test. *p < 0.05 vs. normal control; #*p* < 0.05 vs. D-galactose group.

**Figure 6.**
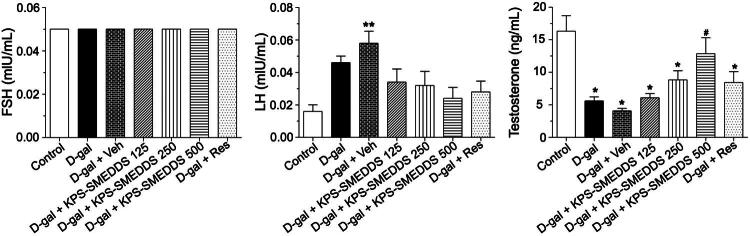
Effect of KPS-SMEDDS on reproductive hormone levels in D-galactose-induced aging rats. Rats were administered D-galactose to induce aging and subsequently treated with KPS-SMEDDS orally at doses of 125, 250, or 500 mg/kg body weight for 60 days. Serum levels of follicle-stimulating hormone (FSH), luteinizing hormone (LH), and testosterone were measured and are presented as mean ± SEM (*n* = 9 per group). Statistical significance was determined using one-way ANOVA followed by Tukey’s post hoc test. *p < 0.05 indicates a significant difference compared with the normal control group, while #*p* < 0.05 indicates a significant difference compared with the D-galactose-induced group.

Although there were no significant differences between groups in the liver enzymes ALT and AST, the groups that received 125 and 250 mg/kg body weight of KPS-SMEDDS showed a downward trend in these enzyme levels compared to the D-gal + Veh group. Interestingly, the lipid profile measurement demonstrated that triglyceride levels in the highest dose-treated group (KPS-SMEDDS 500 mg/kg body weight) were significantly lower than those in the control and D-gal groups. Additionally, LDL levels in the KPS-SMEDDS-treated groups tended to decrease in a dose-dependent manner compared to the untreated aging groups (D-gal and D-gal + Veh) (Figure 5). In addition, LH levels were significantly increased in the D-gal + Veh group compared to the control group. The highest dose-treated group (KPS-SMEDDS 500 mg/kg body weight) showed markedly lower LH levels compared to the untreated aging groups, although levels remained higher than those of the control group. In contrast to LH, testosterone levels were significantly decreased in the untreated aging groups compared to controls (Figure 6).

#### KPS-SMEDDS reduced the D-galactose-induced increase in MDA levels in rats

MDA is a by-product of lipid peroxidation, a process that damages cells and is associated with aging (Angirekula et al. 2018). In this study, MDA levels were measured in various organs. The testis, brain, kidney, and liver of the D-galactose-induced aging group showed significantly higher MDA levels (*p* < 0.05) compared to the control group, confirming successful establishment of the aging model. In contrast, MDA levels in the KPS-SMEDDS-treated groups were significantly lower (*p* < 0.05) than those in the aging model group (Table 4).

**Table 4.** Malondialdehyde (MDA) levels in male D-galactose-induced aging rats following KPS-SMEDDS administration.

Group	MDA (nmol/mL)			
	Testis	Brain	Kidney	Liver
Control	48.06 ± 0.70^#^	23.10 ± 0.56^#^	25.77 ± 0.13^#^	18.02 ± 0.09^#^
D-gal	95.14 ± 1.23*	92.13 ± 1.25*	79.02 ± 0.52*	65.53 ± 0.47*
D-gal + Vehicle	88.56 ± 1.10*	96.90 ± 1.31*	94.55 ± 1.03*	78.42 ± 0.41*
D-gal + KPS-SMEDDS 125	60.75 ± 1.48	19.54 ± 0.23^#^	25.86 ± 0.16^#^	29.95 ± 0.10^#^
D-gal + KPS-SMEDDS 250	32.14 ± 0.22^#^	27.09 ± 0.28^#^	27.85 ± 0.14^#^	30.70 ± 0.12^#^
D-gal + KPS-SMEDDS 500	49.78 ± 0.56^#^	29.68 ± 0.13^#^	43.92 ± 0.60^#^	31.99 ± 0.12^#^
D-gal + Resveratrol	50.51 ± 1.40	26.05 ± 0.28^#^	36.08 ± 0.22^#^	19.93 ± 0.23^#^

The values are expressed as mean ± SEM (*n* = 9 per group). Statistical significance was determined using one-way ANOVA followed by Tukey’s post hoc test. *p < 0.05 indicates a significant difference compared with the control group, while #*p* < 0.05 indicates a significant difference compared with the D-galactose-induced group.

#### KPS-SMEDDS administration counteracted the histopathological alterations of testicular tissue observed in D-galactose-induced aging rats

**Figure 7.**
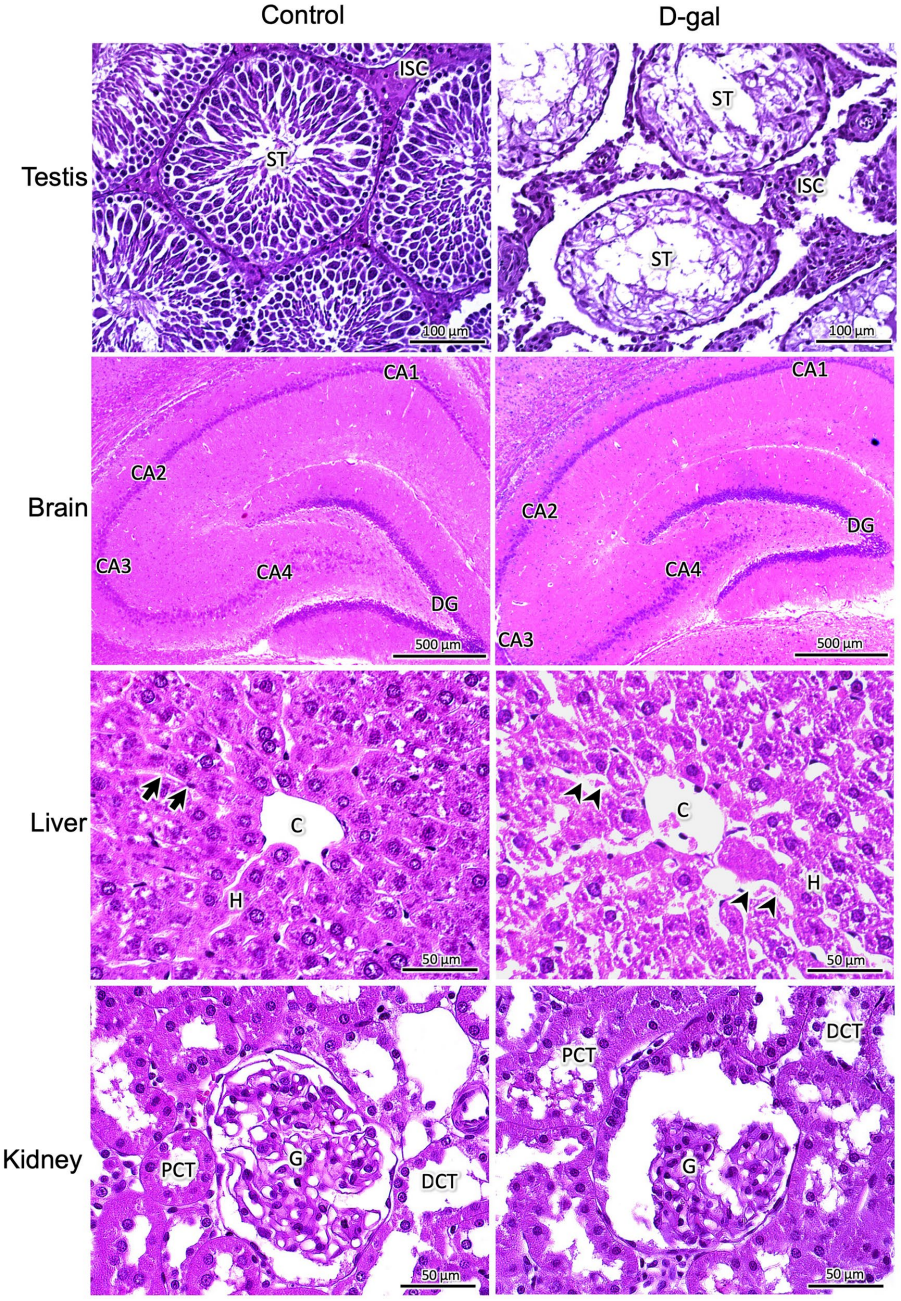
Histological features of the testis, brain, kidney, and liver in control and D-galactose-induced aging rats. Representative light micrographs show tissue architecture in the control and D-galactose-treated groups. In the testis, ST indicates seminiferous tubules and ISC indicates interstitial cells. In the brain, DG represents the dentate gyrus and CA1–4 represent the cornu Ammonis regions of the hippocampus. In the liver, C indicates the central vein, H indicates hepatic cords, arrows denote hepatic sinusoids, and arrowheads indicate dilated sinusoids. In the kidney, G indicates glomeruli, PCT indicates proximal convoluted tubules, and DCT indicates distal convoluted tubules. All tissues were stained with hematoxylin and eosin (H&E) and examined under light microscopy to assess structural changes associated with D-galactose-induced aging. Scale bars = 50 μm, 100 μm, 500 μm.

The results showed that D-galactose induced histopathological changes in various organs, as shown in Figure 7, with the most pronounced alterations observed in the testis. Based on these findings, the testis was selected for further investigation. This decision was supported by the significant reduction in testicular weight observed in the D-galactose-induced group compared to the control, which was reversed following KPS-SMEDDS administration. Additionally, previous studies have reported the impact of D-galactose-induced aging on testicular structure and function (Sulistyoningrum 2017; Lee et al. 2023).

**Figure 8.**
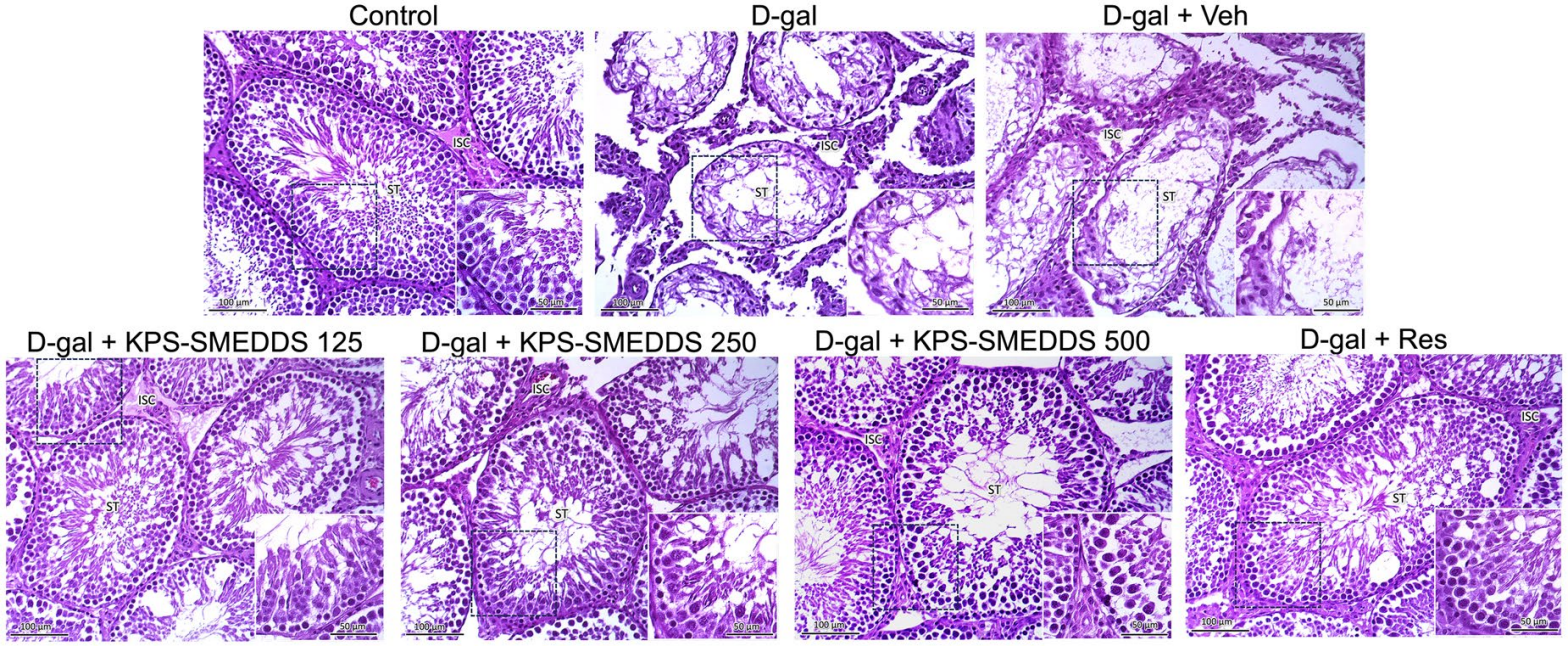
Histological evaluation of the testis in D-galactose-induced aging rats treated with KPS-SMEDDS for 60 days. Representative light micrographs show the testicular tissue architecture in rats receiving KPS-SMEDDS. ST indicates seminiferous tubules, and ISC indicates interstitial cells. Micrographs include both main images (scale bar = 100 μm) and insets highlighting detailed structures (scale bar = 50 μm). All sections were stained with hematoxylin and eosin (H&E) to assess structural changes and the protective effects of KPS-SMEDDS against D-galactose-induced testicular aging.

The morphological features of hematoxylin and eosin-stained testis sections are presented in Figure 8. Testes from the control group exhibited normal histological architecture. Numerous seminiferous tubules were observed, separated by intact interstitial tissue. The tubules appeared rounded or oval and were surrounded by peritubular myoid cells. They were lined with stratified germinal epithelium composed of two distinct cell populations: spermatogenic cells and Sertoli cells. The spermatogenic cells represented various stages of spermatogenesis, including spermatogonia, primary spermatocytes, spermatids, and well-differentiated spermatozoa. Narrow interlobular spaces containing interstitial tissue were present, with clusters of interstitial cells displaying ovoid or polygonal shapes and spherical nuclei. In contrast, testes from the aging model groups (D-gal and D-gal + Veh) showed disruption of normal histological architecture, with varying degrees of seminiferous tubule atrophy. The tubules were separated by widened interlobular spaces containing degenerated interstitial cells. The number of spermatogonia was reduced, and other stages of spermatogenic cells – such as primary spermatocytes, spermatids, and spermatozoa – were rarely observed. Degenerative changes in Sertoli cells were also apparent. Treatment with KPS-SMEDDS attenuated these degenerative changes and restored both the number and morphology of spermatogenic cells within the seminiferous tubules.

#### Ultrastructural changes in D-galactose-induced aging rats were mitigated by KPS-SMEDDS administration

**Figure 9.**
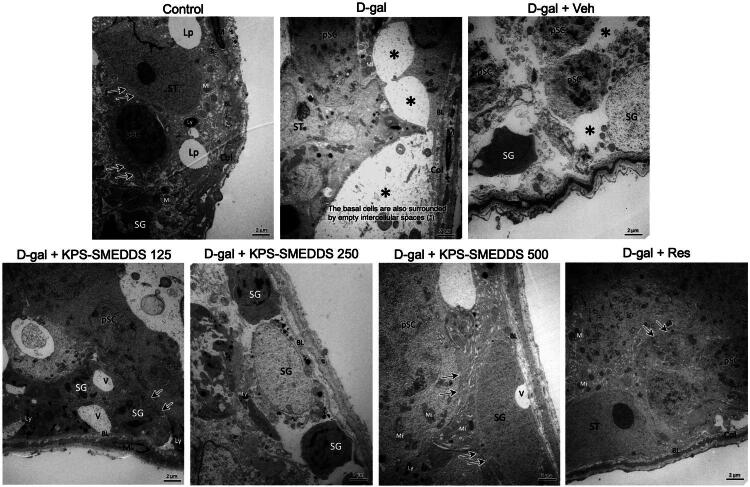
Ultrastructural analysis of testicular tissue in D-galactose-induced aging rats treated with KPS-SMEDDS for 60 days. Transmission electron micrographs show spermatogenic and Sertoli cells within the seminiferous tubules. SG indicates spermatogonia, pSC indicates primary spermatocytes, and ST indicates Sertoli cells. Other features include lipid droplets (Lp), lysosomes (Ly), basal lamina (BL), myoid cells (M), mitochondria (Mi), collagen fibers (Col), vacuoles (V), cell junctions (arrow), and intercellular spaces (asterisk). Scale bar = 2 μm. Images illustrate subcellular structural changes and the protective effects of KPS-SMEDDS against D-galactose-induced testicular aging.

To clarify the effect of KPS-SMEDDS in attenuating degenerative changes and restoring testicular cells in D-galactose-induced aging, transmission electron microscopy (TEM) was performed. As shown in Figure 9, ultrastructural observation of testes from control rats revealed normal testicular architecture. Well-developed Sertoli cells exhibited large, oval nuclei with prominent nucleoli. The cytoplasm of Sertoli cells extended from the basal lamina to the lumen of the seminiferous tubules and enveloped adjacent germinal cells. Primary spermatocytes appeared rounded with prominent nuclei, displaying distinct chromatin networks, well-defined nuclear membranes, and intact cell junctions. Both spermatogenic and Sertoli cells showed a normal peripheral distribution of mitochondria and contained few lysosomes. The seminiferous epithelium was surrounded by a regular basal lamina and a lamina propria composed of collagen fibers and myoid cells. In contrast, the ultrastructure of aged testes in the aging model groups (D-gal and D-gal + Veh) showed numerous germ cells containing lysosomes and residual bodies within the cytoplasm, indicating organelle degeneration. Both spermatogonia and primary spermatocytes exhibited nuclear and cytoplasmic alterations, including loss of nuclear membranes, displacement of nuclei from basal positions, cytoplasmic degeneration, increased vacuolization, and widened intercellular spaces. However, treatment with KPS-SMEDDS at a dose of 500 mg/kg body weight attenuated these degenerative changes. Germ cells remained attached to the basal lamina, contained normal organelles, and exhibited only minimal cytoplasmic vacuolization. The nuclear membrane and cell junctions were restored. Taken together, these findings suggest that KPS-SMEDDS exerts a protective effect against D-galactose-induced testicular aging.

#### Effects of KPS-SMEDDS on the expression of aging-related proteins in D-galactose-induced rats

**Figure 10.**
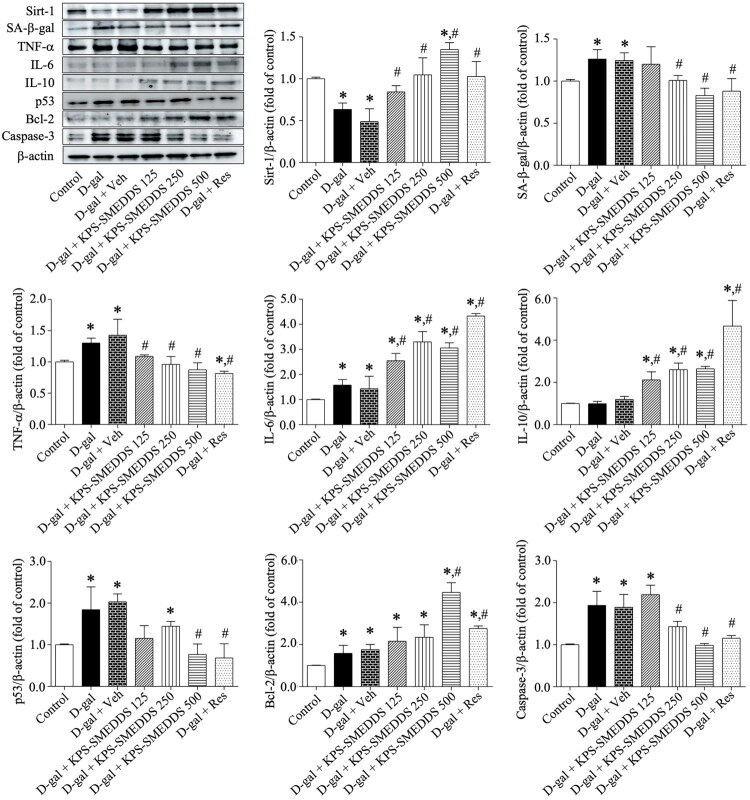
Effect of KPS-SMEDDS on the expression of aging-related proteins in D-galactose-induced aging rats. Representative protein bands and quantitative analysis show the expression of Sirt-1, SA-β-gal, TNF-α, IL-6, IL-10, p53, Bcl-2, and caspase-3 in testicular tissue, normalized to β‑actin. Experimental groups included control group (Control), D-galactose group (D-gal), D-galactose + vehicle (D-gal + Veh), D-galactose + KPS-SMEDDS 125 mg/kg body weight (D-gal + KPS-SMEDDS 125), D-galactose + KPS-SMEDDS 250 mg/kg body weight (D-gal + KPS-SMEDDS 250), D-galactose + KPS-SMEDDS 500 mg/kg body weight (D-gal + KPS-SMEDDS 500), and D-galactose + Resveratrol (D-gal + Res). Protein expression is presented as fold change relative to the control group. Data are expressed as mean ± SEM (*n* = 3) from three independent experiments. Statistical significance was determined using one-way ANOVA followed by Tukey’s post hoc test. *p < 0.05 vs. control group; #*p* < 0.05 vs. D-gal group. These results demonstrate the modulatory effects of KPS-SMEDDS on molecular markers associated with aging and apoptosis.

In this study, we further examined the expression of aging-related proteins in D-galactose-induced rats and KPS-SMEDDS–treated rats. As shown in Figure 10, Western blot analysis revealed that the expression levels of SA-β-gal, TNF-α, IL-6, p53, Bcl-2, and caspase-3 were markedly increased, while the expression of Sirt-1 was decreased in the D-galactose-induced aging model groups (D-gal and D-gal + Veh). Compared with the aging model group, KPS-SMEDDS treatment at doses of 125, 250, and 500 mg/kg body weight significantly reduced the elevated expression of SA-β-gal, TNF-α, IL-6, p53, and caspase-3. In addition to downregulating these aging-related proteins, KPS-SMEDDS also upregulated the expression of Sirt-1, IL-10, and Bcl-2 compared to D-galactose–treated rats. These effects were comparable to those observed in the resveratrol-treated group (D-gal + Res). These findings suggest that the anti-aging effects of KPS-SMEDDS are mediated through the modulation of aging-related protein expression, including markers of aging, inflammation, and apoptosis.

## Discussion

The sub-chronic toxicity study revealed that oral administration of the solid self-microemulsifying drug delivery system of *K. parviflora* extract (KPS-SMEDDS) at doses of 125, 250, and 500 mg/kg body weight did not cause toxicity in either male or female rats, consistent with findings reported by Yoshino et al. (2019). In the present study, KPS-SMEDDS at a dose of 500 mg/kg/day had no effect on body weight in any treatment group after 90 days of administration. This observation aligns with a previous report in which rats receiving *K. parviflora* extract at 500 mg/kg/day for 12 weeks during a chronic toxicity study showed no changes in body weight (Chivapat et al. 2010). Additionally, no overt signs of toxicity or illness were observed in any group. Our study demonstrated a dose-dependent trend toward decreased cholesterol levels in KPS-SMEDDS-treated male rats. Furthermore, the highest dose group exhibited lower triglyceride levels in both males and females compared to their respective control groups. This finding is consistent with a previous report by Somintara et al. (2018), which showed that *K. parviflora* extract reduced triglyceride levels in normal rats after 4 weeks of administration.

KPS-SMEDDS treatment also reduced LDL-cholesterol levels. These findings are consistent with previous results showing that oral administration of *K. parviflora* extract at daily doses of 150 or 300 mg/kg body weight for 12 weeks significantly reduced cholesterol, triglyceride, and LDL levels in dyslipidemic rats (Somintara et al. 2019). Similarly, a study by Akase et al. (2011) found that *K. parviflora* extract decreased total cholesterol and triglyceride levels in Tsumura Suzuki Obese Diabetes (TSOD) mice. Furthermore, a randomized, double-blind, placebo-controlled clinical trial in overweight and pre-obese Japanese adults demonstrated that daily intake of *K. parviflora* extract (150 mg/day) significantly lowered triglyceride levels and reduced visceral fat, subcutaneous fat, and total abdominal fat areas, as assessed by CT scans. In this study, the cholesterol and LDL-cholesterol–lowering effects of KPS-SMEDDS were evident only in male rats, indicating a sex-specific response consistent with known biological differences in lipid metabolism. Male rodents typically exhibit higher baseline hepatic cholesterol synthesis and greater activity of key lipogenic enzymes, making them more responsive to lipid-lowering interventions (Link et al. 2015). Sex hormones also strongly influence lipid homeostasis: testosterone is associated with increased LDL-cholesterol levels, whereas estrogen promotes LDL receptor expression and enhances hepatic clearance of circulating LDL (Björnström and Sjöberg 2005; Palmisano et al. 2018). As a result, female rodents naturally maintain a more favorable lipid profile, which may diminish the measurable effects of KPS-SMEDDS. Participants in the extract group also showed decreased body weight and body fat percentage compared to the placebo group (Yoshino et al. 2018). Consistent with our lipid findings, improved delivery of *K. parviflora* polymethoxyflavones (PMFs) can plausibly translate into better control of lipid homeostasis through several complementary mechanisms. First, nanocarrier formulations of *K. parviflora* methoxyflavones enhance gastrointestinal stability, intestinal permeability, and systemic exposure, and directly suppress adipocyte lipid accumulation and triglyceride production; *in vivo*, these effects accompany reductions in adiposity in high-fat-diet models, supporting a primary anti-adipogenic/antilipogenic action when oral bioavailability is optimized (Rimsueb et al. 2025). Second, PMFs-rich extracts activate cellular energy-sensing pathways – most notably Sirt-1/AMPK and downstream PGC-1α/PPAR programs – which are known to inhibit hepatic de novo lipogenesis, promote fatty-acid oxidation, and improve lipid profiles (Kim et al. 2018; Lee et al. 2018). Third, *K. parviflora*’s antioxidant and anti-inflammatory activities (including Nrf2-ARE pathway engagement) may indirectly normalize lipid handling by attenuating oxidative stress and inflammatory signaling that drive dyslipidemia (Huang et al. 2024). Taken together, we posit that KPS-SMEDDS augments methoxyflavone exposure, thereby amplifying AMPK/PPAR-centered metabolic reprogramming and anti-adipogenic effects, which mechanistically explains the observed reductions in LDL-cholesterol, triglyceride, and total cholesterol. Consistently, no changes in HbA1c levels were observed in any male rats receiving KPS-SMEDDS. In contrast, a previous study reported elevated glucose levels in female rats treated with the highest dose of *K. parviflora* rhizome powder (Chivapat et al. 2010). However, in the present study, no changes in glucose levels were observed in female rats. This discrepancy may be attributed to differences in chemical composition and concentrations of active constituents between *K. parviflora* rhizome powder and KPS-SMEDDS.

In addition to the findings in male rats, no treatment-related abnormalities were observed in females. Ovarian histology showed normal follicular development, intact corpora lutea, and the absence of inflammatory or degenerative changes following 90-day administration of KPS-SMEDDS, indicating no detectable reproductive toxicity. Hematological profiles in female rats also remained within physiological limits across all dose groups, consistent with the absence of systemic adverse effects. These results parallel the safety profile observed in males and suggest that KPS-SMEDDS is well tolerated in both sexes under sub-chronic exposure. Nonetheless, further studies assessing reproductive hormones and estrous cycle patterns would be valuable to clarify potential sex-specific responses.

Aging is influenced by various factors, including lifestyle, nutrition, genetics, and physical activity (Hosseini et al. 2021). It can result in permanent alterations in multiple organs, including the male reproductive system, potentially leading to male infertility (Hussein et al. 2020). Proposed mechanisms underlying aging include oxidative stress, mitochondrial dysfunction, inflammation, and apoptosis (Tao et al. 2024). The present study focused on evaluating the anti-aging efficacy of KPS-SMEDDS in a D-galactose-induced aging model.

Generally, aging models are classified into two categories: natural aging and accelerated aging (Harkema et al. 2016). Due to practical considerations, such as the length and duration of animal care, the D-galactose-induced accelerated aging model was selected. Prolonged exposure to D-galactose leads to excessive production of reactive oxygen species (ROS), resulting in redox system imbalance, oxidative stress, and ultimately the induction of aging (Li et al. 2024a). Numerous studies have shown that subchronic or chronic administration of D-galactose can effectively model testicular aging (Aydin et al. 2018; Lee et al. 2023). D-galactose induces aging-related changes, including disorganization of testicular architecture, reduced sperm count and antioxidant capacity, increased free radical production, and immune dysfunction (Ahangarpour et al. 2014).

We found that exposure to D-galactose altered testicular weight, as well as the levels of luteinizing hormone (LH), testosterone, and malondialdehyde (MDA) in rats. In the untreated aging groups, testosterone levels declined significantly compared to the controls, whereas LH levels increased (Figure 6). This hormonal pattern aligns with well-documented age-related dysregulation of the hypothalamic–pituitary–gonadal (HPG) axis, in which reduced testicular testosterone production leads to diminished androgen-mediated negative feedback at both the hypothalamus and anterior pituitary (Veldhuis et al. 2009; Cheng et al. 2024). Under normal physiology, low circulating testosterone stimulates gonadotropin-releasing hormone (GnRH) secretion from the hypothalamus, which subsequently increases pituitary LH release in an effort to restore testosterone synthesis by Leydig cells. However, with aging, the testes – particularly Leydig cells – exhibit impaired responsiveness to LH due to reduced steroidogenic capacity, mitochondrial dysfunction, and decreased expression of steroidogenic enzymes (Chen et al. 2009; Zirkin and Tenover 2012). This results in a persistent state of elevated LH but low testosterone, characteristic of primary hypogonadism in older males. Notably, treatment with D-galactose with KPS-SMEDDS at 500 mg/kg significantly increased testosterone levels, suggesting an improvement in Leydig cell function and a partial restoration of negative feedback regulation within the HPG axis, which may explain the normalization of LH levels. It also affected the structure of the seminiferous tubules and the morphology of germ cells, changes comparable to those observed in senescent rats. In contrast, the weights of other organs (brain, kidney, and liver) and blood biochemical parameters – including ALT, AST, BUN, creatinine, cholesterol, triglycerides, HDL, LDL, and FSH – were not significantly affected. These differences may be attributed to variations in the route of administration, dosage, and duration of D-galactose exposure (Sulistyoningrum 2017). For example, intraperitoneal injection of D-galactose at 200 mg/kg body weight for 8 weeks increased serum MDA levels and reduced testicular weight and sperm parameters (Liao et al. 2016). Oral gavage of D-galactose at 3 mg/kg body weight for 6 weeks decreased sperm parameters and testosterone levels, while increasing lactate dehydrogenase (LDH), MDA, and LH levels (Salman et al. 2016). Subcutaneous injection of 5% D-galactose resulted in reduced sperm count and relative organ weights, along with histological changes in the testis, epididymis, and seminal vesicles (Shaikh et al. 2015).

The alterations caused by D-galactose treatment in rats were mitigated by KPS-SMEDDS administration in this study, indicating the potential of this compound in anti-aging activity. Natural products like KPS-SMEDDS have been recognized as anti-aging agents capable of promoting health and extending lifespan in animal models (Chen et al. 2022). For example, epigallocatechin-3-gallate (EGCG) has been shown to prolong the lifespan of high-fat diet–induced obese rats by improving free fatty acid metabolism, reducing inflammation, and alleviating oxidative stress (Yuan et al. 2020). Similarly, an alkaloid from *Sophora flavescens* attenuated D-galactose–induced age-related cognitive impairment by inhibiting cellular senescence and oxidative stress (Sun et al. 2018).

MDA, a by-product formed during the final stages of lipid peroxidation initiated by excessive ROS, is widely regarded as a biomarker of aging (Tsikas et al. 2023). Supplementation with antioxidants has been reported to slow the aging process (Koyama et al. 2013). In the present study, oral administration of KPS-SMEDDS attenuated MDA levels in the testis, brain, kidney, and liver. These findings support both the proposed mechanism of action of KPS-SMEDDS and the oxidative stress theory of aging. Flavonoid activity has been shown to improve spermatogenesis by reducing oxidative stress and enhancing testosterone production in the testis (Zhang et al. 2023). Because testicular antioxidant activity, apoptosis, and cell proliferation are regulated by testosterone (Aly 2021; Khan et al. 2024), and our study showed reduced testosterone levels in D-galactose–induced aged rats, it may be hypothesized that KPS-SMEDDS-mediated testosterone synthesis contributes to the regulation of anti-apoptotic activity and the maintenance of antioxidant defense in the aging testis. Furthermore, the observed changes in luteinizing hormone (LH) levels may be explained by the feedback regulation of the hypothalamic-pituitary-gonadal axis (Veldhuis 2008).

To date, no reliable and independent aging biomarkers exist that can precisely reflect an individual’s aging status or predict the aging process and lifespan. However, several cellular aging biomarkers have been documented (Tao et al. 2024). Sirtuin-1 (Sirt-1) is a nicotinamide adenine dinucleotide (NAD^+^)–dependent deacetylase involved in numerous cellular processes linked to aging and age-related diseases (Herranz et al. 2010). Overexpression of Sirt-1 has been shown to extend lifespan in yeast, worms, flies, and mice, identifying it as a promising pharmacological target for anti-aging interventions and the prevention of age-related disorders (Chen et al. 2020; Rogina and Tissenbaum 2024). Additionally, senescence-associated β-galactosidase (SA-β-gal) is commonly used to identify senescent cells, particularly those undergoing replicative senescence or age-related functional decline (Gao et al. 2024). Elevated SA-β-gal activity in cells and tissues is indicative of cellular senescence (Morgunova et al. 2015). In the present study, D-galactose treatment decreased Sirt-1 expression and increased SA-β-gal activity, while KPS-SMEDDS administration reversed these changes – demonstrating increased Sirt-1 expression and decreased SA-β-gal activity – supporting its anti-aging efficacy.

Many studies have demonstrated that chronic inflammatory responses activate the nuclear factor (NF)-κB signaling pathway, a key intracellular cascade involved in inflammation that drives tissue cells toward senescence (Li et al. 2024b). Activation of the NF-κB pathway upregulates the expression of pro-inflammatory cytokines such as TNF-α and IL-6, which act in opposition to the anti-inflammatory cytokine IL-10 (Saraiva et al. 2020). In addition, inflammatory mediators can promote cellular aging by upregulating p53 expression, which plays a central role in modulating cellular senescence (Bourgeois and Madl 2018). In the present study, D-galactose administration significantly increased the expression of pro-inflammatory cytokines (TNF-α and IL-6) and the senescence-associated marker p53, while reducing the anti-inflammatory cytokine IL-10. Treatment with KPS-SMEDDS effectively attenuated the D-galactose–induced elevations in TNF-α, p53, SA-β-gal, and caspase-3, supporting the notion that KPS-SMEDDS mitigates cellular senescence and apoptosis. These findings parallel the reported anti-aging properties of resveratrol, which reduces pro-inflammatory mediators and enhances anti-inflammatory cytokines (Uddin et al. 2021), as well as ginsenoside Rb1, which has been shown to ameliorate age-related physiological alterations and downregulate p53 expression in aging tissues (Yu et al. 2020).

Interestingly, we observed an increase in IL-6 expression in KPS-SMEDDS–treated animals compared with the D-galactose group (Figure 10). This observation does not necessarily contradict the overall anti-aging profile, as IL-6 is a pleiotropic cytokine with context- and pathway-specific roles. Classical IL-6 signaling *via* membrane-bound IL-6R is commonly associated with tissue repair, regeneration, and anti-inflammatory actions, whereas IL-6 trans-signaling mediated through soluble IL-6R promotes chronic inflammation (Scheller et al. 2011; Hunter and Jones 2015). A plausible explanation is that KPS-SMEDDS may induce a regenerative or early-phase IL-6 response that facilitates repair processes and may subsequently enhance anti-inflammatory pathways, such as IL-10 production, while concurrently suppressing chronic inflammatory mediators like TNF-α and apoptosis-related markers. Thus, a transient or pathway-specific increase in IL-6 may still contribute to an overall protective outcome.

Apoptosis increases with advancing age in the testes of various mammals, and caspase-3 is a key executioner protease involved in germ cell apoptosis (Zakariah et al. 2022). In this study, D-galactose treatment induced apoptosis, whereas KPS-SMEDDS administration markedly upregulated the expression of Bcl-2 (anti-apoptotic) and downregulated the expression of caspase-3 (pro-apoptotic) in the testis compared to the aging group. These findings are consistent with previous reports showing increased apoptosis in testicular germ cells with age, supporting the concept that aging negatively impacts sperm DNA integrity (Kaltsas et al. 2023). With age, the enzymatic defense mechanisms that protect the germinal epithelium become inadequate to counteract oxidative damage, resulting in increased apoptotic events, particularly in germ cells. Consequently, aged testes undergo profound histological and morphological changes that impair testicular function (Matzkin et al. 2016). In our study, electron micrographs revealed that treatment with KPS-SMEDDS at a dose of 500 mg/kg body weight attenuated D-galactose–induced testicular degeneration. Germ cells remained attached to the basal lamina, displayed normal organelles, and contained only a few cytoplasmic vacuoles. The nuclear membrane was clearly defined, and cell junctions were intact. Despite these promising findings, this study has several limitations. Only a single animal model was used, the duration of treatment was limited to 60–90 days, and not all organs or aging-related pathways were evaluated. Future studies should include additional models, longer treatment periods, and a broader assessment of systemic effects to fully establish the anti-aging potential of KPS-SMEDDS.

## Conclusions

This study demonstrates that KPS-SMEDDS is a safe and well-tolerated formulation of *K. parviflora* in rats, with no adverse effects observed in sub-chronic toxicity assessments. Additionally, KPS-SMEDDS exhibited protective effects against D-galactose-induced aging by reducing oxidative stress, modulating inflammatory and apoptotic pathways, and restoring reproductive hormone levels and testicular structure. These results suggest that KPS-SMEDDS has potential as a natural anti-aging agent and provide a foundation for future clinical studies.

## Supplementary Material

Supplemental Material

## Data Availability

The datasets used and/or analyzed during the current study are available from the corresponding author on reasonable request.
