# Supplementary material for: Assessment of sub-chronic toxicity and anti-aging effects of a solid self-microemulsifying drug delivery system of Kaempferia parviflora extract in a D-galactose-induced rat model
Source: Pharm Biol. 2026 Jan 5;64(1):143–67. doi: 10.1080/13880209.2025.2606956 (PMC12777754; doi:10.1080/13880209.2025.2606956)
Supplement: Supplemental Material [file IPHB_A_2606956_SM3125.zip › Figure S1_Caption.docx]

**Figure S1.** Histopathological assessment of major organs in male and female rats treated with a high dose of KPS-SMEDDS (500 mg/kg body weight) for 90 days. Representative photomicrographs of liver, kidney, spleen, brain, heart, lung, stomach, intestine, testis, and ovary were stained with hematoxylin and eosin (H&E) to evaluate potential tissue alterations following chronic oral administration. Key structures are labeled as follows: A, arteriole; AL, alveoli; AS, alveolar sac; C, central vein; CA, corpus albicans; CA1–CA4, cornu Ammonis 1–4; Ch, chief cells; CL, corpus luteum; DG, dentate gyrus; E, epicardium; F, follicle; G, glomerulus; H, hepatic cord; He, helicine arteries; M, myocardium; MM, muscularis mucosae; DCT, distal convoluted tubule; PCT, proximal convoluted tubule; P, parietal cells; RP, red pulp; ST, seminiferous tubule; WP, white pulp; V, intestinal villi; arrowheads, hepatic sinusoid; arrows, epicardium. These images demonstrate that high-dose KPS-SMEDDS did not induce observable histopathological changes in the examined organs. Scale bars = 50 μm, 100 μm, 500 μm.
